# Di-μ-nicotinamide-κ^2^ *N* ^1^:*O*;κ^2^ *O*:*N* ^1^-bis­[aqua­bis­(4-bromo­benzoato)-κ*O*;κ^2^ *O*,*O*′-manganese(II)]

**DOI:** 10.1107/S1600536811028492

**Published:** 2011-07-23

**Authors:** Hacali Necefoğlu, Füreya Elif Özbek, Vijdan Öztürk, Vedat Adıgüzel, Tuncer Hökelek

**Affiliations:** a Kafkas University, Department of Chemistry, 36100 Kars, Turkey; b Hacettepe University, Department of Physics, 06800 Beytepe, Ankara, Turkey

## Abstract

In the centrosymmetric dinuclear title compound, [Mn_2_(C_7_H_4_BrO_2_)_4_(C_6_H_6_N_2_O)_2_(H_2_O)_2_], the Mn^II^ atom is coordinated by one N atom from one bridging nicotinamide ligand and one O atom from another symmetry-related bridging nicotinamide ligand, three O atoms from two 4-bromo­benzoate ligands and one water mol­ecule in a distorted octa­hedral geometry. The dihedral angles between the carboxyl­ate groups and the adjacent benzene rings are 10.89 (16) and 8.4 (2)°, while the benzene rings are oriented at a dihedral angle of 6.09 (13)°. Inter­molecular O—H⋯O, N—H⋯O and weak C—H⋯O hydrogen bonds link the mol­ecules into a three-dimensional network. π–π inter­actions, indicated by short centroid–centroid distances [3.845 (2) Å between the benzene rings, 3.650 (2) Å between the pyridine rings and 3.700 (3) Å between the benzene and pyridine rings] further stabilize the structure.

## Related literature

For niacin, see: Krishnamachari (1974 ▶). For information on the nicotinic acid derivative *N*,*N*-diethyl­nicotinamide, see: Bigoli *et al.* (1972 ▶). For related structures, see: Hökelek *et al.* (2009*a* ▶,*b* ▶); Hökelek *et al.* (2010*a* ▶,*b* ▶); Necefoğlu *et al.* (2011 ▶); Greenaway *et al.* (1984 ▶).
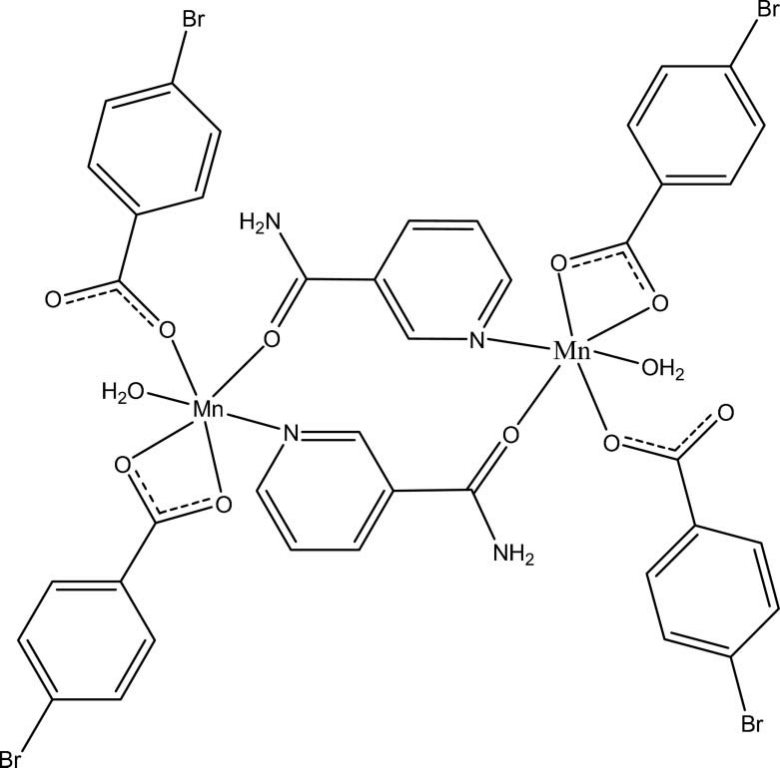


## Experimental

### 

#### Crystal data

[Mn_2_(C_7_H_4_BrO_2_)_4_(C_6_H_6_N_2_O)_2_(H_2_O)_2_]
*M* *_r_* = 1190.18
Triclinic, 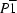

*a* = 7.2213 (2) Å
*b* = 12.1782 (3) Å
*c* = 13.4931 (4) Å
α = 110.038 (3)°
β = 91.206 (2)°
γ = 103.653 (2)°
*V* = 1076.51 (6) Å^3^
*Z* = 1
Mo *K*α radiation
μ = 4.37 mm^−1^
*T* = 294 K
0.29 × 0.22 × 0.20 mm

#### Data collection

Bruker Kappa APEXII CCD area-detector diffractometer
Absorption correction: multi-scan (*SADABS*; Bruker, 2005 ▶) *T*_min_ = 0.329, *T*_max_ = 0.418
18469 measured reflections
5394 independent reflections
4298 reflections with *I* > 2σ(*I*)
*R*_int_ = 0.039

#### Refinement

*R*[*F* ^2^ > 2σ(*F* ^2^)] = 0.049
*wR*(*F* ^2^) = 0.126
*S* = 1.05
5394 reflections
297 parameters
4 restraints
H atoms treated by a mixture of independent and constrained refinement
Δρ_max_ = 2.03 e Å^−3^
Δρ_min_ = −1.76 e Å^−3^

### 

Data collection: *APEX2* (Bruker, 2007 ▶); cell refinement: *SAINT* (Bruker, 2007 ▶); data reduction: *SAINT*; program(s) used to solve structure: *SHELXS97* (Sheldrick, 2008 ▶); program(s) used to refine structure: *SHELXL97* (Sheldrick, 2008 ▶); molecular graphics: *ORTEP-3 for Windows* (Farrugia, 1997 ▶); software used to prepare material for publication: *WinGX* (Farrugia, 1999 ▶) and *PLATON* (Spek, 2009 ▶).

## Supplementary Material

Crystal structure: contains datablock(s) I, global. DOI: 10.1107/S1600536811028492/xu5272sup1.cif

Structure factors: contains datablock(s) I. DOI: 10.1107/S1600536811028492/xu5272Isup2.hkl

Additional supplementary materials:  crystallographic information; 3D view; checkCIF report

## Figures and Tables

**Table 1:** Selected bond lengths (Å)

Mn1—O1	2.316 (2)
Mn1—O2	2.237 (2)
Mn1—O3	2.025 (3)
Mn1—O5	2.217 (2)
Mn1—O6	2.170 (2)
Mn1—N1	2.272 (3)

**Table 2:** Hydrogen-bond geometry (Å, °)

*D*—H⋯*A*	*D*—H	H⋯*A*	*D*⋯*A*	*D*—H⋯*A*
N2—H2*A*⋯O2^i^	0.85 (3)	2.02 (3)	2.856 (4)	168 (5)
O6—H6*A*⋯O1^ii^	0.84 (5)	1.96 (6)	2.771 (4)	162 (5)
O6—H6*B*⋯O4^ii^	0.84 (4)	1.83 (4)	2.670 (4)	175 (4)
C17—H17⋯O2^iii^	0.93	2.27	3.125 (5)	152

Symmetry codes: (i) 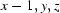; (ii) 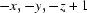; (iii) 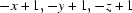.

**Fig. 1.**
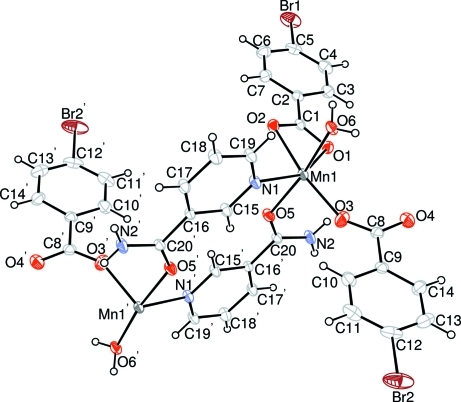
The molecular structure of the title molecule with the atom-numbering scheme. Displacement ellipsoids are drawn at the 50% probability level [symmetry code: (') -x, -y, -z].

**Fig. 2.**
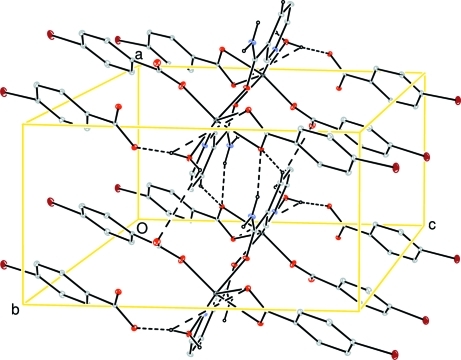
A view of the crystal packing of the title compound. Hydrogen bonds are shown as dashed lines. [H-atoms not involved in hydrogen bonding have been omitted for clarity].

## supplementary crystallographic information

### Comment

As a part of our ongoing investigations of transition metal complexes of nicotinamide (NA), one form of niacin (Krishnamachari, 1974), and/or the nicotinic acid derivative *N*,*N*-diethylnicotinamide (DENA), an important respiratory stimulant (Bigoli *et al.*, 1972), the title compound was synthesized and its crystal structure is reported herein.

The title compound, (I), consists of dimeric units located around a crystallographic symmetry centre and made up of two Mn cations, four 4-bromobenzoate (PBB) anions, which act in different modes-monodentate, bidentate and bidentate, monodentate, respectively, two nicotinamide (NA) ligands and two water molecules (Fig. 1). Both of the Mn^II^ centres are six-coordinated, and the two monomeric units are bridged through the two nicotinamide (NA) ligands about an inversion center. The Mn1···Mn1^i^ [symmetry code: (i) -x, -y, -z] distance is 7.180 (2) Å. In the molecule, one Mn—O bond distance [2.316 (2) Å] is significantly longer than the other four, and the average Mn—O bond length is 2.193 (2) Å (Table 1). The Mn atom is displaced out of the least-squares planes of the carboxylate groups (O1/C1/O2) and (O3/C8/O4) by -0.0925 (5) and -0.5744 (5) Å, respectively.

The dihedral angles between the planar carboxylate groups and the adjacent benzene rings A (C2—C7) and B (C9—C14) are 10.89 (16) and 8.37 (20) °, respectively, while those between rings A, B and C (N1/C15—C19) are A/B = 6.09 (13), A/C = 85.37 (11), B/C = 86.41 (13) °.

In (I), the O1-Mn1-O2 angle is 57.61 (8)°. The corresponding O—M—O (where M is a metal) angles are 57.75 (2)° in [Cu(C_7_H_5_O_2_F)(C_7_H_4_O_2_F)_2_(C_6_H_6_N_2_O)_2_], (II) (Necefoğlu *et al.*, 2011), 60.32 (4)° in [Co(C_8_H_7_O_3_)_2_(C_6_H_6_N_2_O)(H_2_O)_2_], (III) (Hökelek *et al.*, 2010*a*), 59.02 (8)° in [Zn(C_8_H_8_NO_2_)_2_(C_6_H_6_N_2_O)_2_].H_2_O, (IV) (Hökelek *et al.*, 2009*a*), 60.03 (6)° in [Zn(C_9_H_10_NO_2_)_2_(C_6_H_6_N_2_O)_2_(H_2_O)_2_], (V) (Hökelek *et al.*, 2009*b*), 57.53 (5)°, 56.19 (5)° and 59.04 (4)° in [Zn(C_8_H_7_O_3_)_2_(C_6_H_6_N_2_O)_2_], (VI) (Hökelek *et al.*, 2010*b*) and 55.2 (1)° in [Cu(Asp)_2_(py)_2_] (where Asp is acetylsalicylate and py is pyridine) [(VII); Greenaway *et al.*, 1984].

In the crystal structure, intermolecular O—H···O, N—H···O and C—H···O hydrogen bonds link the molecules into a three dimensional network (Table 2, Fig. 2), in which they may be effective in the stabilization of the structure. The π–π contacts between the benzene rings, between the pyridine rings and the benzene and pyridine rings Cg1—Cg1^i^, Cg3—Cg3^ii^ and Cg1—Cg2^iii^ [symmetry codes: (i) 2 - x, -y, -z; (ii) 3 - x, 1 - y, 1 - z; (iii) 2 - x, -y, 1 - z, where Cg1, Cg2 and Cg3 are the centroids of the rings A (C2—C7), B (C9—C14) and C (N1/C15—C19), respectively] may further stabilize the structure, with centroid-centroid distances of 3.845 (2), 3.650 (2) and 3.700 (3) Å, respectively.

### Experimental

The title compound was prepared by the reaction of MnSO_4_.H_2_O (0.85 g, 10 mmol) in H_2_O (25 ml) and nicotinamide (1.22 g, 20 mmol) in H_2_O (25 ml) with sodium 4-bromobenzoate (2.23 g, 20 mmol) in H_2_O (100 ml) at room temperature. The mixture was filtered and set aside to crystallize at ambient temperature for a few days, giving colorless single crystals.

### Refinement

Atoms H6A, H6B (for H_2_O) and H2A, H2B (for NH_2_) were located in a difference Fourier map and were freely refined. The C-bound H-atoms were positioned geometrically with C—H = 0.93 Å for aromatic H-atoms, and constrained to ride on their parent atoms, with *U*_iso_(H) = *1.2U*_eq_(C).

### Crystal data

**Table:**

[Mn_2_(C_7_H_4_BrO_2_)_4_(C_6_H_6_N_2_O)_2_(H_2_O)_2_]	*Z* = 1
*M**_r_* = 1190.18	*F*(000) = 586
Triclinic, *P*1	*D*_x_ = 1.836 Mg m^−^^3^
Hall symbol: -P 1	Mo *K*α radiation, λ = 0.71073 Å
*a* = 7.2213 (2) Å	Cell parameters from 7870 reflections
*b* = 12.1782 (3) Å	θ = 2.9–28.1°
*c* = 13.4931 (4) Å	µ = 4.37 mm^−^^1^
α = 110.038 (3)°	*T* = 294 K
β = 91.206 (2)°	Block, colorless
γ = 103.653 (2)°	0.29 × 0.22 × 0.20 mm
*V* = 1076.51 (6) Å^3^	

### Data collection

**Table:**

Bruker Kappa APEXII CCD area-detector diffractometer	5394 independent reflections
Radiation source: fine-focus sealed tube	4298 reflections with *I* > 2σ(*I*)
graphite	*R*_int_ = 0.039
φ and ω scans	θ_max_ = 28.6°, θ_min_ = 2.0°
Absorption correction: multi-scan (*SADABS*; Bruker, 2005)	*h* = −9→9
*T*_min_ = 0.329, *T*_max_ = 0.418	*k* = −16→16
18469 measured reflections	*l* = −18→17

### Refinement

**Table:**

Refinement on *F*^2^	Secondary atom site location: difference Fourier map
Least-squares matrix: full	Hydrogen site location: inferred from neighbouring sites
*R*[*F*^2^ > 2σ(*F*^2^)] = 0.049	H atoms treated by a mixture of independent and constrained refinement
*wR*(*F*^2^) = 0.126	*w* = 1/[σ^2^(*F*_o_^2^) + (0.0463*P*)^2^ + 2.4514*P*] where *P* = (*F*_o_^2^ + 2*F*_c_^2^)/3
*S* = 1.05	(Δ/σ)_max_ < 0.001
5394 reflections	Δρ_max_ = 2.03 e Å^−^^3^
297 parameters	Δρ_min_ = −1.76 e Å^−^^3^
4 restraints	Extinction correction: *SHELXL97* (Sheldrick, 2008), Fc^*^=kFc[1+0.001xFc^2^λ^3^/sin(2θ)]^-1/4^
Primary atom site location: structure-invariant direct methods	Extinction coefficient: 0.0235 (13)

### Special details

**Table:**

Geometry. All esds (except the esd in the dihedral angle between two l.s. planes) are estimated using the full covariance matrix. The cell esds are taken into account individually in the estimation of esds in distances, angles and torsion angles; correlations between esds in cell parameters are only used when they are defined by crystal symmetry. An approximate (isotropic) treatment of cell esds is used for estimating esds involving l.s. planes.
Refinement. Refinement of F^2^ against ALL reflections. The weighted R-factor wR and goodness of fit S are based on F^2^, conventional R-factors R are based on F, with F set to zero for negative F^2^. The threshold expression of F^2^ > 2sigma(F^2^) is used only for calculating R-factors(gt) etc. and is not relevant to the choice of reflections for refinement. R-factors based on F^2^ are statistically about twice as large as those based on F, and R- factors based on ALL data will be even larger.

### Fractional atomic coordinates and isotropic or equivalent isotropic displacement parameters (Å^2^)

**Table:**

	*x*	*y*	*z*	*U*_iso_*/*U*_eq_	
Mn1	0.03281 (7)	0.20681 (4)	0.48986 (4)	0.02469 (14)	
Br1	0.08035 (10)	−0.25888 (5)	−0.16236 (4)	0.0704 (2)	
Br2	−0.35117 (11)	0.46846 (7)	1.09799 (4)	0.0875 (3)	
O1	−0.0971 (3)	0.0349 (2)	0.34227 (19)	0.0305 (5)	
O2	0.1724 (3)	0.1626 (2)	0.33974 (19)	0.0294 (5)	
O3	−0.1656 (5)	0.1988 (3)	0.5927 (3)	0.0537 (8)	
O4	−0.3453 (4)	0.0368 (2)	0.6157 (2)	0.0470 (7)	
O5	−0.1222 (3)	0.3095 (2)	0.4261 (2)	0.0320 (5)	
O6	0.2150 (4)	0.1154 (2)	0.5439 (2)	0.0326 (5)	
H6A	0.174 (7)	0.083 (5)	0.588 (3)	0.068 (17)*	
H6B	0.259 (6)	0.066 (3)	0.496 (3)	0.042 (12)*	
N1	0.2516 (4)	0.3847 (2)	0.5796 (2)	0.0291 (6)	
N2	−0.4244 (4)	0.1935 (3)	0.3759 (3)	0.0369 (7)	
H2A	−0.541 (3)	0.187 (4)	0.357 (4)	0.054 (14)*	
H2B	−0.394 (7)	0.133 (3)	0.384 (4)	0.053 (13)*	
C1	0.0442 (4)	0.0650 (3)	0.2952 (2)	0.0243 (6)	
C2	0.0602 (5)	−0.0145 (3)	0.1855 (3)	0.0281 (7)	
C3	−0.0629 (5)	−0.1295 (3)	0.1423 (3)	0.0358 (8)	
H3	−0.1523	−0.1571	0.1827	0.043*	
C4	−0.0537 (6)	−0.2035 (4)	0.0392 (3)	0.0439 (9)	
H4	−0.1349	−0.2811	0.0106	0.053*	
C5	0.0761 (6)	−0.1610 (4)	−0.0197 (3)	0.0433 (9)	
C6	0.2021 (6)	−0.0479 (4)	0.0219 (3)	0.0453 (9)	
H6	0.2915	−0.0212	−0.0190	0.054*	
C7	0.1942 (5)	0.0256 (3)	0.1250 (3)	0.0354 (8)	
H7	0.2789	0.1021	0.1538	0.043*	
C8	−0.2679 (5)	0.1464 (3)	0.6458 (3)	0.0318 (7)	
C9	−0.2941 (5)	0.2254 (3)	0.7544 (3)	0.0314 (7)	
C10	−0.1872 (6)	0.3452 (3)	0.7977 (3)	0.0412 (9)	
H10	−0.1026	0.3769	0.7574	0.049*	
C11	−0.2037 (7)	0.4181 (4)	0.8993 (4)	0.0515 (11)	
H11	−0.1332	0.4986	0.9273	0.062*	
C12	−0.3271 (7)	0.3688 (4)	0.9580 (3)	0.0512 (11)	
C13	−0.4343 (6)	0.2518 (5)	0.9188 (4)	0.0522 (11)	
H13	−0.5171	0.2207	0.9602	0.063*	
C14	−0.4181 (6)	0.1794 (4)	0.8158 (3)	0.0425 (9)	
H14	−0.4911	0.0995	0.7881	0.051*	
C15	0.2168 (5)	0.4876 (3)	0.5798 (3)	0.0291 (7)	
H15	0.0945	0.4845	0.5539	0.035*	
C16	0.3514 (4)	0.5991 (3)	0.6163 (2)	0.0235 (6)	
C17	0.5302 (5)	0.6036 (3)	0.6579 (3)	0.0313 (7)	
H17	0.6249	0.6762	0.6834	0.038*	
C18	0.5661 (5)	0.4984 (3)	0.6609 (3)	0.0373 (8)	
H18	0.6852	0.4998	0.6897	0.045*	
C19	0.4250 (5)	0.3911 (3)	0.6212 (3)	0.0325 (7)	
H19	0.4515	0.3208	0.6234	0.039*	
C20	−0.2925 (4)	0.2957 (3)	0.3966 (3)	0.0252 (6)	

### Atomic displacement parameters (Å^2^)

**Table:**

	*U*^11^	*U*^22^	*U*^33^	*U*^12^	*U*^13^	*U*^23^
Mn1	0.0273 (2)	0.0187 (2)	0.0281 (3)	0.00505 (17)	0.00231 (18)	0.00905 (18)
Br1	0.1104 (5)	0.0750 (4)	0.0318 (2)	0.0543 (3)	0.0100 (2)	0.0063 (2)
Br2	0.1231 (6)	0.1180 (5)	0.0361 (3)	0.0877 (5)	0.0081 (3)	0.0083 (3)
O1	0.0304 (12)	0.0269 (11)	0.0325 (12)	0.0038 (9)	0.0061 (10)	0.0109 (9)
O2	0.0269 (11)	0.0266 (11)	0.0337 (12)	0.0043 (9)	0.0009 (9)	0.0114 (9)
O3	0.0640 (19)	0.0517 (17)	0.0500 (18)	0.0148 (15)	0.0300 (15)	0.0226 (14)
O4	0.0580 (18)	0.0320 (13)	0.0502 (17)	0.0133 (12)	0.0130 (14)	0.0123 (12)
O5	0.0243 (11)	0.0230 (10)	0.0482 (15)	0.0044 (9)	−0.0054 (10)	0.0138 (10)
O6	0.0429 (14)	0.0239 (11)	0.0354 (14)	0.0116 (10)	0.0044 (11)	0.0141 (10)
N1	0.0297 (14)	0.0208 (12)	0.0361 (15)	0.0041 (10)	−0.0034 (11)	0.0113 (11)
N2	0.0276 (15)	0.0221 (13)	0.060 (2)	0.0016 (11)	−0.0041 (14)	0.0175 (14)
C1	0.0263 (15)	0.0230 (13)	0.0264 (15)	0.0076 (11)	−0.0012 (12)	0.0116 (12)
C2	0.0300 (16)	0.0301 (15)	0.0274 (16)	0.0121 (13)	0.0007 (13)	0.0117 (13)
C3	0.0403 (19)	0.0303 (16)	0.0323 (18)	0.0080 (14)	0.0012 (15)	0.0065 (14)
C4	0.054 (2)	0.0332 (18)	0.038 (2)	0.0134 (17)	−0.0039 (17)	0.0047 (16)
C5	0.060 (2)	0.047 (2)	0.0288 (18)	0.034 (2)	0.0022 (17)	0.0079 (16)
C6	0.050 (2)	0.060 (3)	0.039 (2)	0.027 (2)	0.0165 (18)	0.0250 (19)
C7	0.0354 (18)	0.0396 (18)	0.0342 (19)	0.0113 (15)	0.0048 (14)	0.0157 (15)
C8	0.0300 (16)	0.0357 (17)	0.0366 (18)	0.0163 (14)	0.0073 (14)	0.0161 (14)
C9	0.0319 (17)	0.0359 (17)	0.0351 (18)	0.0183 (14)	0.0065 (14)	0.0171 (14)
C10	0.049 (2)	0.0363 (19)	0.042 (2)	0.0151 (16)	0.0051 (17)	0.0163 (16)
C11	0.070 (3)	0.040 (2)	0.044 (2)	0.025 (2)	−0.003 (2)	0.0081 (18)
C12	0.064 (3)	0.064 (3)	0.033 (2)	0.046 (2)	0.0022 (19)	0.0071 (19)
C13	0.045 (2)	0.079 (3)	0.047 (2)	0.031 (2)	0.0176 (19)	0.030 (2)
C14	0.038 (2)	0.050 (2)	0.045 (2)	0.0143 (17)	0.0127 (17)	0.0219 (18)
C15	0.0259 (15)	0.0221 (14)	0.0378 (18)	0.0033 (12)	−0.0062 (13)	0.0112 (13)
C16	0.0248 (14)	0.0196 (13)	0.0263 (15)	0.0047 (11)	0.0018 (11)	0.0092 (11)
C17	0.0259 (15)	0.0255 (15)	0.0408 (19)	0.0019 (12)	−0.0053 (13)	0.0134 (14)
C18	0.0289 (17)	0.0336 (17)	0.051 (2)	0.0064 (14)	−0.0079 (15)	0.0190 (16)
C19	0.0361 (18)	0.0255 (15)	0.0391 (19)	0.0096 (13)	−0.0021 (14)	0.0148 (14)
C20	0.0262 (15)	0.0196 (13)	0.0278 (15)	0.0053 (11)	0.0008 (12)	0.0064 (11)

### Geometric parameters (Å, °)

**Table:**

Mn1—O1	2.316 (2)	C6—C5	1.377 (6)
Mn1—O2	2.237 (2)	C6—H6	0.9300
Mn1—O3	2.025 (3)	C7—C6	1.384 (6)
Mn1—C1	2.619 (3)	C7—H7	0.9300
Br1—C5	1.891 (4)	C9—C8	1.497 (5)
Br2—C12	1.901 (4)	C9—C10	1.389 (5)
O1—C1	1.260 (4)	C9—C14	1.384 (5)
O2—C1	1.265 (4)	C10—C11	1.380 (6)
O3—C8	1.257 (4)	C10—H10	0.9300
O4—C8	1.235 (4)	C11—H11	0.9300
Mn1—O5	2.217 (2)	C12—C13	1.362 (7)
O5—C20	1.239 (4)	C12—C11	1.373 (7)
Mn1—O6	2.170 (2)	C13—H13	0.9300
O6—H6A	0.845 (19)	C14—C13	1.392 (6)
O6—H6B	0.847 (19)	C14—H14	0.9300
Mn1—N1	2.272 (3)	C15—C16	1.389 (4)
N1—C15	1.334 (4)	C15—H15	0.9300
N1—C19	1.335 (4)	C16—C17	1.378 (4)
N2—H2A	0.853 (19)	C16—C20^i^	1.503 (4)
N2—H2B	0.857 (19)	C17—H17	0.9300
C1—C2	1.490 (5)	C18—C17	1.380 (5)
C2—C3	1.386 (5)	C18—H18	0.9300
C2—C7	1.388 (5)	C19—C18	1.379 (5)
C3—C4	1.388 (5)	C19—H19	0.9300
C3—H3	0.9300	C20—N2	1.314 (4)
C4—H4	0.9300	C20—C16^i^	1.503 (4)
C5—C4	1.364 (6)		
			
O1—Mn1—C1	28.77 (9)	C4—C5—Br1	119.1 (3)
O2—Mn1—O1	57.61 (8)	C4—C5—C6	121.6 (4)
O2—Mn1—N1	96.47 (10)	C6—C5—Br1	119.3 (3)
O2—Mn1—C1	28.85 (9)	C5—C6—C7	119.3 (4)
O3—Mn1—O1	102.93 (11)	C5—C6—H6	120.3
O3—Mn1—O2	160.32 (12)	C7—C6—H6	120.3
O3—Mn1—O5	89.12 (12)	C2—C7—H7	120.0
O3—Mn1—O6	97.50 (12)	C6—C7—C2	120.1 (4)
O3—Mn1—N1	102.86 (12)	C6—C7—H7	120.0
O3—Mn1—C1	131.68 (12)	O3—C8—C9	116.0 (3)
O5—Mn1—O1	89.54 (9)	O4—C8—O3	125.3 (4)
O5—Mn1—O2	87.89 (9)	O4—C8—C9	118.7 (3)
O5—Mn1—N1	87.93 (9)	C10—C9—C8	120.6 (3)
O5—Mn1—C1	89.17 (9)	C14—C9—C8	120.8 (3)
O6—Mn1—O1	91.37 (9)	C14—C9—C10	118.5 (4)
O6—Mn1—O2	86.68 (9)	C9—C10—H10	119.3
O6—Mn1—O5	172.93 (10)	C11—C10—C9	121.4 (4)
O6—Mn1—N1	88.18 (10)	C11—C10—H10	119.3
O6—Mn1—C1	88.26 (10)	C10—C11—H11	120.9
N1—Mn1—O1	154.04 (10)	C12—C11—C10	118.2 (4)
N1—Mn1—C1	125.32 (10)	C12—C11—H11	120.9
C1—O1—Mn1	89.05 (18)	C11—C12—Br2	118.6 (4)
C1—O2—Mn1	92.58 (19)	C13—C12—Br2	119.1 (4)
C8—O3—Mn1	152.3 (3)	C13—C12—C11	122.3 (4)
C20—O5—Mn1	134.3 (2)	C12—C13—C14	119.0 (4)
Mn1—O6—H6B	116 (3)	C12—C13—H13	120.5
Mn1—O6—H6A	117 (4)	C14—C13—H13	120.5
H6B—O6—H6A	108 (5)	C9—C14—C13	120.5 (4)
C15—N1—Mn1	118.9 (2)	C9—C14—H14	119.7
C15—N1—C19	117.4 (3)	C13—C14—H14	119.7
C19—N1—Mn1	123.1 (2)	N1—C15—C16	124.2 (3)
C20—N2—H2A	121 (3)	N1—C15—H15	117.9
C20—N2—H2B	120 (3)	C16—C15—H15	117.9
H2A—N2—H2B	119 (5)	C15—C16—C20^i^	117.2 (3)
O1—C1—Mn1	62.18 (17)	C17—C16—C15	117.5 (3)
O1—C1—O2	120.7 (3)	C17—C16—C20^i^	125.2 (3)
O1—C1—C2	119.9 (3)	C16—C17—C18	118.8 (3)
O2—C1—Mn1	58.57 (17)	C16—C17—H17	120.6
O2—C1—C2	119.4 (3)	C18—C17—H17	120.6
C2—C1—Mn1	177.4 (2)	C17—C18—H18	120.1
C3—C2—C1	119.6 (3)	C19—C18—C17	119.9 (3)
C3—C2—C7	119.4 (3)	C19—C18—H18	120.1
C7—C2—C1	121.0 (3)	N1—C19—C18	122.2 (3)
C2—C3—C4	120.4 (4)	N1—C19—H19	118.9
C2—C3—H3	119.8	C18—C19—H19	118.9
C4—C3—H3	119.8	O5—C20—N2	123.2 (3)
C3—C4—H4	120.4	O5—C20—C16^i^	118.5 (3)
C5—C4—C3	119.1 (4)	N2—C20—C16^i^	118.3 (3)
C5—C4—H4	120.4		
			
O2—Mn1—O1—C1	1.32 (17)	C19—N1—Mn1—O1	−78.8 (4)
O3—Mn1—O1—C1	178.1 (2)	C19—N1—Mn1—O2	−75.9 (3)
O3—Mn1—O2—C1	−10.6 (4)	C19—N1—Mn1—O3	107.9 (3)
O5—Mn1—O1—C1	89.11 (19)	C19—N1—Mn1—O5	−163.5 (3)
O6—Mn1—O1—C1	−83.88 (19)	C19—N1—Mn1—O6	10.6 (3)
N1—Mn1—O1—C1	4.8 (3)	C19—N1—Mn1—C1	−76.0 (3)
O1—Mn1—O2—C1	−1.31 (17)	Mn1—N1—C15—C16	−168.9 (3)
O5—Mn1—O2—C1	−92.11 (18)	C19—N1—C15—C16	2.8 (5)
O6—Mn1—O2—C1	92.42 (18)	Mn1—N1—C19—C18	169.8 (3)
N1—Mn1—O2—C1	−179.79 (18)	C15—N1—C19—C18	−1.5 (6)
O1—Mn1—O3—C8	56.2 (7)	O1—C1—C2—C3	−10.1 (5)
O2—Mn1—O3—C8	64.3 (8)	O2—C1—C2—C3	170.3 (3)
O5—Mn1—O3—C8	145.5 (7)	O1—C1—C2—C7	168.6 (3)
O6—Mn1—O3—C8	−37.0 (7)	O2—C1—C2—C7	−10.9 (5)
N1—Mn1—O3—C8	−126.8 (6)	C1—C2—C3—C4	178.2 (3)
C1—Mn1—O3—C8	57.4 (7)	C7—C2—C3—C4	−0.6 (5)
O1—Mn1—C1—O2	177.7 (3)	C1—C2—C7—C6	−177.6 (3)
O2—Mn1—C1—O1	−177.7 (3)	C3—C2—C7—C6	1.2 (5)
O3—Mn1—C1—O1	−2.5 (3)	C2—C3—C4—C5	−1.0 (6)
O3—Mn1—C1—O2	175.22 (19)	Br1—C5—C4—C3	−177.0 (3)
O5—Mn1—C1—O1	−90.56 (18)	C6—C5—C4—C3	2.0 (6)
O5—Mn1—C1—O2	87.14 (18)	C7—C6—C5—Br1	177.6 (3)
O6—Mn1—C1—O1	96.03 (19)	C7—C6—C5—C4	−1.5 (6)
O6—Mn1—C1—O2	−86.28 (18)	C2—C7—C6—C5	−0.2 (6)
N1—Mn1—C1—O1	−177.43 (17)	C10—C9—C8—O3	−9.3 (5)
N1—Mn1—C1—O2	0.3 (2)	C10—C9—C8—O4	170.2 (3)
Mn1—O1—C1—O2	−2.3 (3)	C14—C9—C8—O3	173.9 (4)
Mn1—O1—C1—C2	178.2 (3)	C14—C9—C8—O4	−6.5 (5)
Mn1—O2—C1—O1	2.4 (3)	C8—C9—C10—C11	−177.4 (4)
Mn1—O2—C1—C2	−178.1 (2)	C14—C9—C10—C11	−0.6 (6)
Mn1—O3—C8—O4	−37.6 (9)	C8—C9—C14—C13	176.7 (4)
Mn1—O3—C8—C9	142.0 (5)	C10—C9—C14—C13	−0.2 (6)
C20—O5—Mn1—O1	55.0 (3)	C9—C10—C11—C12	1.1 (6)
C20—O5—Mn1—O2	112.6 (3)	Br2—C12—C11—C10	−180.0 (3)
C20—O5—Mn1—O3	−47.9 (3)	C13—C12—C11—C10	−0.9 (7)
C20—O5—Mn1—N1	−150.8 (3)	Br2—C12—C13—C14	179.2 (3)
C20—O5—Mn1—C1	83.8 (3)	C11—C12—C13—C14	0.2 (7)
Mn1—O5—C20—N2	−15.3 (5)	C9—C14—C13—C12	0.3 (6)
Mn1—O5—C20—C16^i^	164.6 (2)	N1—C15—C16—C17	−2.1 (5)
C15—N1—Mn1—O1	92.4 (3)	N1—C15—C16—C20^i^	175.6 (3)
C15—N1—Mn1—O2	95.3 (3)	C15—C16—C17—C18	0.2 (5)
C15—N1—Mn1—O3	−80.9 (3)	C20^i^—C16—C17—C18	−177.4 (3)
C15—N1—Mn1—O5	7.7 (3)	C19—C18—C17—C16	1.0 (6)
C15—N1—Mn1—O6	−178.2 (3)	N1—C19—C18—C17	−0.3 (6)
C15—N1—Mn1—C1	95.2 (3)		

Symmetry codes: (i) −*x*, −*y*+1, −*z*+1.

### Hydrogen-bond geometry (Å, °)

**Table:**

*D*—H···*A*	*D*—H	H···*A*	*D*···*A*	*D*—H···*A*
N2—H2A···O2^ii^	0.85 (3)	2.02 (3)	2.856 (4)	168 (5)
O6—H6A···O1^iii^	0.84 (5)	1.96 (6)	2.771 (4)	162 (5)
O6—H6B···O4^iii^	0.84 (4)	1.83 (4)	2.670 (4)	175 (4)
C15—H15···O5	0.93	2.40	3.032 (4)	125
C17—H17···O2^iv^	0.93	2.27	3.125 (5)	152
C19—H19···O6	0.93	2.53	3.129 (5)	123

Symmetry codes: (ii) *x*−1, *y*, *z*; (iii) −*x*, −*y*, −*z*+1; (iv) −*x*+1, −*y*+1, −*z*+1.
